# Effects of Ethanol and Acetaldehyde on Tight Junction Integrity: In Vitro Study in a Three Dimensional Intestinal Epithelial Cell Culture Model

**DOI:** 10.1371/journal.pone.0035008

**Published:** 2012-04-19

**Authors:** Elhaseen Elamin, Daisy Jonkers, Kati Juuti-Uusitalo, Sven van IJzendoorn, Freddy Troost, Hans Duimel, Jos Broers, Fons Verheyen, Jan Dekker, Ad Masclee

**Affiliations:** 1 Top Institute Food and Nutrition, Wageningen, The Netherlands; 2 Division of Gastroenterology-Hepatology, Maastricht University Medical Center, Maastricht, The Netherlands; 3 School for Nutrition, Toxicology and Metabolism, Maastricht University Medical Center, Maastricht, The Netherlands; 4 Department of Cell Biology, University Medical Center Groningen, Groningen, The Netherlands; 5 Department of Molecular Cell Biology, Maastricht University Medical Center, Maastricht, The Netherlands; 6 Electron Microscopy Unit, Maastricht University Medical Center, Maastricht, The Netherlands; 7 Department of Animal Sciences, Wageningen University, Wageningen, The Netherlands; University of Pittsburgh, United States of America

## Abstract

**Background:**

Intestinal barrier dysfunction and translocation of endotoxins are involved in the pathogenesis of alcoholic liver disease. Exposure to ethanol and its metabolite, acetaldehyde at relatively high concentrations have been shown to disrupt intestinal epithelial tight junctions in the conventional two dimensional cell culture models. The present study investigated quantitatively and qualitatively the effects of ethanol at concentrations detected in the blood after moderate ethanol consumption, of its metabolite acetaldehyde and of the combination of both compounds on intestinal barrier function in a three-dimensional cell culture model.

**Methods and Findings:**

Caco-2 cells were grown in a basement membrane matrix (Matrigel™) to induce spheroid formation and were then exposed to the compounds at the basolateral side. Morphological differentiation of the spheroids was assessed by immunocytochemistry and transmission electron microscopy. The barrier function was assessed by the flux of FITC-labeled dextran from the basal side into the spheroids' luminal compartment using confocal microscopy. Caco-2 cells grown on Matrigel assembled into fully differentiated and polarized spheroids with a central lumen, closely resembling enterocytes *in vivo* and provide an excellent model to study epithelial barrier functionality. Exposure to ethanol (10–40 mM) or acetaldehyde (25–200 µM) for 3 h, dose-dependently and additively increased the paracellular permeability and induced redistribution of ZO-1 and occludin without affecting cell viability or tight junction-encoding gene expression. Furthermore, ethanol and acetaldehyde induced lysine residue and microtubules hyperacetylation.

**Conclusions:**

These results indicate that ethanol at concentrations found in the blood after moderate drinking and acetaldehyde, alone and in combination, can increase the intestinal epithelial permeability. The data also point to the involvement of protein hyperacetylation in ethanol- and acetaldehyde-induced loss of tight junctions integrity.

## Introduction

It is well known that consumption of ethanol may result in a variety of noxious effects in the human body, especially in the liver [1]. Previous studies in humans and in animal models have demonstrated that ethanol causes intestinal mucosa damage, which may give rise to an increase in intestinal permeability [2], [3], [4], [5], [6], [7]. This allows harmful and potentially toxic luminal compounds, such as endotoxins to enter the systemic circulation and to contribute to alcoholic liver disease or alcohol related diseases in other organs [4], [8], [9], [10], [11]. Intestinal mucosal barrier function and integrity depend on an intact paracellular pathway, which is largely regulated by intercellular junctions, i.e. tight junctions (TJs), adherens junctions (AJs) and desmosomes [12]. The TJs are multiprotein complexes composed of transmembrane proteins (occludin, the claudin family, tricellulin, junction adhesion molecule, and others) that interact with the cytoplasmic plaque proteins (e.g., ZO-1, ZO-2, ZO-3, AF6, cingulin), which in turn interact with F-actin to anchor occludin and the other transmembrane proteins to the cytoskeleton perijunctional ring of actomyosin [12], [13], [14], [15], [16], [17], [18], [19], [20]. *In vitro* studies using the conventional two dimensional (2D) cell culture model of intestinal cell monolayers grown on filters have shown that ethanol as well as its main metabolite, acetaldehyde disrupt epithelial TJs integrity and thereby increase paracellular permeability [21], [22], [23], [24], [25], [26], [27], [28]. Recently, it has been reported that ethanol synergizes acetaldehyde-induced TJs disruption [29]. The *in vitro* data published so far have shown that the disruption of intestinal barrier function occurs after apical exposure to ethanol in concentrations at 1% v/v (∼217 mM) and higher [21], [22], [26], [27], [28]. Such concentrations can only be found in the lumen of the small intestine, immediately after “binge drinking”, which is defined as consumption of least 160 g/day [30]. Although variations have been reported worldwide in the definition of moderate ethanol consumption, consumption of (12–24 g/day) on a regular base is more widespread [31]. This amount of up to 24 g/day can result in ethanol serum concentrations of about 10–40 mM; one magnitude lower than previously tested [32]. Data on effects of ethanol at these concentrations on intestinal permeability and TJs complex are scarce. Current *in vitro* approaches to study the integrity and permeability of intestinal epithelial monolayers predominantly employ cell culture systems in which epithelial cells are grown on flat Transwell filter membranes. Although such 2-D cell cultures may produce tight epithelial cell monolayers, important microenvironmental conditions that in a coordinated manner promote key signaling pathways and enable cell proliferation, differentiation and monolayer permeability, are lost. As a consequence, 2-D cell culture systems fail to capture physiologically-significant and three-dimensional aspects of tissue biology [33], [34]. Many of these aspects can be secured when cells are cultured in 3-D matrices. This has been demonstrated for epithelial cells of various origins, including intestinal epithelial cells [34], [35], [36], [37], [38], [39], [40], [41]. Thus, unlike conventional 2-D intestinal cell monolayers, intestinal epithelial cells cultured in a 3-D matrix maintain specific morphological and biochemical properties of the in vivo tissue, including formation of microvilli and expression of brush border enzymes and remain in a differentiated and functionally active state for longer periods [42]. Three dimensional intestinal epithelial cultures thus provide an excellent model system to study intestinal epithelial integrity [42].

Aim of the present study was to investigate in the 3D intestinal epithelial cell culture model of Caco-2 cells, the effects of ethanol at concentrations that are found in the blood after moderate drinking, and of the main ethanol metabolite acetaldehyde, on a) intestinal epithelial barrier function b) TJ proteins and c) TJ encoding gene expression.

## Materials and Methods

### Cell line and Culture Conditions

Colonic adenocarcinoma cell line (Caco-2) from the American Type Culture Collection, (ATCC, Rockville, USA) were cultured in Dulbecco's Modified Eagle Medium (DMEM; Lonza Benelux BV, Breda, NL) containing 4.5 g/l glucose and L-glutamine, 10% (v/v) fetal calf serum (Invitrogen, Breda, the Netherlands), 1% (v/v) solution of non-essential amino acids (Invitrogen) and 1% (v/v) solution of antibiotic/antimycotic mixture (10,000 units of penicillin, 10,000 µg of streptomycin, and 25 µg of amphotericin B per ml; Invitrogen) at 37°C and in air plus 5% CO_2_ atmosphere.

### Three Dimensional Epithelial Cell Culture and Exposure to Ethanol and Acetaldehyde

Caco-2 cells were initially grown as standard monolayers on plastic support until they reached approximately 70–80% confluency. Twenty µl growth factor-reduced Matrigel® (8 mg/ml; BD Biosciences, San Jose, California USA) was allowed to solidify at 37°C for 30 minutes in glass bottom culture dishes (MatTek Corporation, Ashland, USA) for barrier function and immunofluorescence analysis, and in 96 well-plates (Corning BV, Amsterdam, the Netherlands) for redox state, mitochondrial function and cell viability assays. The Caco-2 cells (50×10^3^ cells/well; passage 39–48) were resuspended in serum-free medium, mixed with 40% (v/v) Matrigel and plated on the solidified Matrigel. Thereafter, the complete growth medium was added and spheroids were allowed to form over 5–7 days at 37°C. The quality of cultures was checked by counting the number of spheroids from four different quadrants and by classifying them according to the number of lumens formed. Only cultures consisting of more than 70% spheroids with a single lumen were used for further experiments. Spheroids were exposed to ethanol (10 mM, 20 mM and 40 mM), acetaldehyde (25 µM, 50 µM, 100 µM, 200 µM) and the combination of both for 3 h. These concentrations of ethanol are in the range found in the blood after moderate drinking [43]. To prevent evaporation, cultures were kept in small boxes and sealed with a plastic tape. Two mM ethylene glycol tetra acetic acid (EGTA) to induce maximum TJs disruption and growth medium only were used as positive and negative controls, respectively. In separate experiments, spheroids were exposed to Trichostatin A (300 ng/ml), an acetylating agent, for immunofluorescence analysis of protein acetylation.

### Assessment of Intestinal Epithelial Barrier Function

To assess epithelial paracellular barrier function, spheroids were incubated under the above mentioned experimental conditions in the presence of 1 mg/ml fluorescein isothothiocyanate (FITC)-labeled dextran of 4 kDa (FD4; Sigma Chemical Co, Amsterdam, the Netherlands) for 3 h. Although less physiological and being a limitation of the current model, the intestinal barrier function was assessed from the basal to the luminal side, due to difficulties in exposing the luminal side of the model to a test substrate. The flux of FD4 from the basolateral compartment into the lumen was monitored using a Leica TCS SPE confocal laser scanning microscope, equipped with a 63× oil immersion objective (Leica Microsystems GmbH, Mannheim, Germany). The mean fluorescence intensity of the FD4 from 8 spheroids was measured using Image J software and expressed as the ratio of the luminal over the basal compartment, as previously described [42].

### Fluorescent Assessment of Cell Viability

In this test a single-reagent fluorescence assay measures the relative number of living cells in the cell population, based on a single marker for cell viability (CellTiter-Apotox™ Cell Viability Assay, Promega, Amsterdam, the Netherlands). Briefly, when the fluorogenic cell-permeant peptide substrate, glycyl-phenylalanyl-amino-fluorocoumarin (GF-AFC), is cleaved by the live-cell protease activity it releases AFC generating a fluorescent signal proportional to the number of living cells. This live-cell protease activity marker becomes inactive upon loss of cell membrane integrity and leakage to the surrounding culture medium. After culturing the Caco-2 cells in 3D in 96-well plates, the wells were washed twice with HBSS (pH 7.4) and then incubated with 100 µl of either (10–40 mM) ethanol or (25–200 µM) acetaldehyde at 37°C for 3 h. Thereafter, 100 µl of the reagent assay (GF-AFC) was added to each well and incubated for 60 min. Positive (9% Triton X-100 solution) and negative (plain medium) control wells were also used for comparison of maximal and minimal membrane disruption, respectively. The fluorescence was measured at an excitation and emission wavelength of 400 and 505 nm, respectively, by Varioskan Flash (Thermo Fisher Scientific Inc., Waltham, MA, USA). The cell viability was calculated from the fluorescent values and expressed as percentage of the negative control (plain medium only).

### Immunofluorescence Labeling

At the end of the experimental period, Caco-2 spheroids in culture dishes were fixed in 4% (w/v) paraformaldehyde in Hank's Buffered Salt Solution (HBSS; Invitrogen) at 37°C for 40 min. Then, spheroids were washed thrice with HBSS and permeabilized with 0.1% (v/v) Triton X-100 in PBS at room temperature for 40 min. Next, spheroids were incubated with a blocking buffer containing 3% (w/v) bovine serum albumin (BSA) in PBS, pH 7.4, at 37°C for 2 h and washed with HBSS. Thereafter, spheroids were incubated with the following primary antibodies at 1∶100 dilution in 3% (w/v) BSA in PBS, pH 7.4 at 4°C overnight: mouse anti-ZO-1 (Zymed Laboratories, San Francisco, USA), rabbit anti-occludin (Zymed Laboratories), rabbit anti-β-catenin (Abcam, Cambridge, UK), mouse anti-E-cadherin (Abcam), mouse anti-acetylated α-tubulin (Abcam) or rabbit anti-acetylated lysine (Enzo life sciences, Antwerp Belgium). The next morning, spheroids were warmed at room temperature for 20 min, washed thrice with HBSS and incubated with either Cy3-conjugated goat anti rabbit immunoglobulin (Jackson Laboratories, Suffolk, UK) or Alexa-488 conjugated goat anti mouse immunoglobulin (Invitrogen) secondary antibodies (1∶100 dilution in 3% (w/v) BSA in PBS, pH 7.4) at 37°C for 1.5 h. Actin filaments were stained using phalloidin (1∶500 in PBS; Sigma Chemical Co). After another washing in HBSS, spheroids were stained for 5 min with diamidino-2-phenylindole (DAPI; 1∶10,000 dilution in PBS; Sigma Chemical Co). Finally, spheroids were mounted in dishes using VectaShield mounting medium (Vector Laboratories, Burlingame, USA). Confocal images were obtained using a Leica TCS SPE confocal laser scanning microscope (Leica Microsystems GmbH). Image J software was used to process and analyze the images.

### Transmission Electron Microscopy (TEM)

To assess the ultrastructural morphology of TJ and other cell-cell junctions in control spheroids and those exposed to ethanol (10 mM, 20 mM and 40 mM) and acetaldehyde (25 µM, 50 µM, 100 µM, 200 µM) for 3 h, the 3D spheroids were fixed in either 2.5% or Karnovsky's fixative in 0.1 M phosphate buffer (pH 7.4) for 24 hours. After a short rinse in PBS, the samples were postfixed in 1% osmium tetroxide in 0.1 M sodium cacodylate buffer containing 1.5% ferricyanide (pH 7.4, 4°C) for 1 h. Next, the spheroids were rinsed in PBS for 45 min, dehydrated in a graded series of ethanol (70–100%) and routinely embedded in epon (Ladd Research Industries Williston, USA). For light microscopy, 1 µm thin sections were prepared and stained with toluidine blue (TB; Merck, Darmstadt, Germany). Finally, ultrathin sections were cut, stained with uranyl acetate (TED PELLA, Redding, CA) and lead citrate (Acros organics, Geel, Belgium), and examined in a Philips CM100 electron microscope (Philips CM100, Eindhoven, the Netherlands).

### RNA Isolation and Reverse Transcription

Spheroids for RNA isolation were incubated with Matrisperse (BD Biosciences) to digest the Matrigel at 4°C for 2 h. Thereafter, cultures were transferred to Eppendorf tubes and incubated with 1 ml Trizol (Invitrogen), 10 µl β-mercaptoethanol and 200 µl chloroform for 3 min at room temperature. Next, samples were centrifuged at 12,000 rpm for 15 minutes at 4°C. About 600 µl from the upper, colorless phase was mixed with an equal volume of isopropanol and glycogen (20 mg/ml). Then, samples were incubated for 1 h at −20°C and centrifuged at 12,000 rpm at 4°C for 30 min, after which a cell pellet could be seen. The pellets were washed thrice with 75% (v/v) ethanol and dissolved in 20 µl RNase-free water. Quantity and purity of the RNA samples was determined using a Nanodrop spectrophotometer (NanoDrop Technologies, Wilmington, USA).

### Quantification of Gene Expression Using Real-Time PCR

The mRNA levels of claudin-2, claudin-4, ZO-1, occludin, and myosin light chain kinase (MLCK) were assessed by real-time PCR. Briefly, 0.1 µg/µl total RNA was used as a template for the cDNA synthesis using the iScript cDNA synthesis kits (Bio-Rad, Veenendaal, the Netherlands). The cDNA was diluted 40× with RNAse free water. Each reaction contained 12.5 µl iQ Sybr Green Supermix, 1 µl of 10 µM gene-specific forward and reverse primers, 4 µl diluted cDNA template and 5.5 µl sterile H_2_O. As ‘housekeeping’ gene, glyceraldehyde 3-phosphate dehydrogenase (GAPDH) was used. Primer sequences were designed using Beacon designer software and are listed in table 1. Reactions were run on My IQ Single Color Real Time PCR Detection System (Bio-Rad). RT-PCR conditions used were 3 min at 95°C, followed by 40 amplification cycles of 10 seconds at 95°C and 45 seconds at 60°C. Expression of the TJ-coding mRNA was normalized with GAPDH mRNA expression.

**Table 1:** Primer Sequences for Real Time PCR.

Gene	Sequence ID	Forward primer	Reverse primer
GAPDH	NM_002046.3	TGCACCACCAACTGCTTAGC	GGCATGGACTGTGGTCATGAG
CLDN 2	NM_016675.4	ATGGCCTCTCTTGGCCTCCAA	TCACACATACCCTGTCAGGCT
CLDN 4	NM_001305.3	ACAGACAAGCCTTACTCC	GGAAGAACAAAGCAGAG
MLCK	NM_053025.3	GCCTGACCACGAATATAA	GCTCCTTCTCATCATCATC
OCLN	NM_002538.2	TCAGGGAATATCCACCTATCACTTCAG	CATCAGCAGCAGCCATGTACTCTTCAC
ZO-1/TJP-1	NM_003257.3	AGGGGCAGTGGTGGTTTTCTGTTCTTTC	GCAGAGGTCAAAGTTCAAGGCTCAAGAGG

**GADPH: glyceraldehyde-3-phosphate dehydrogenase; CLDN 2: claudin 2; CLDN 4: claudin 4; MLCK: myosin light chain kinase; OCLN: occludin; ZO-1: zonula occludens 1/tight junction protein 1.**

### Statistical Analysis

All experiments were performed in triplicate and results were reported as mean±SD of at least 8 spheroids per experiments. A one-way analysis of variance (ANOVA) and Tukey's *post hoc* test were performed to determine significant differences between experimental conditions. Differences were considered statistically significant when *P*<0.05.

## Results

### Intestinal Epithelial Caco-2 Cells in 3D Culture

**Figure 1:**
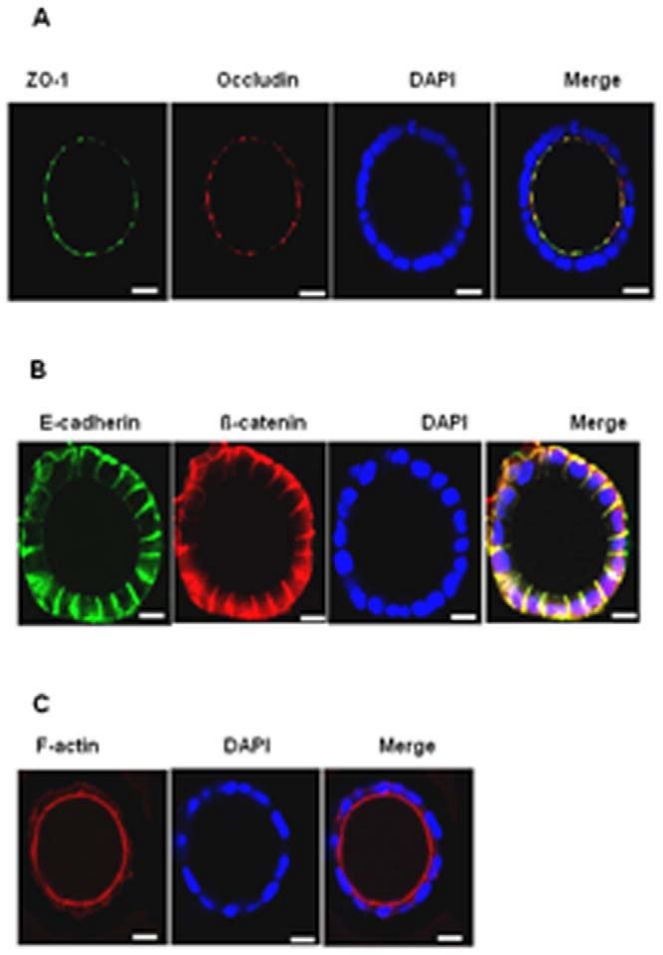
Three-dimensional Caco-2 spheroids express the TJ, the AJ proteins and the peri-junctional actin ring. [A] Protein expression of ZO-1 and occludin was detected using immunofluorescence microscopy (original magnification, ×63) with mouse anti- ZO-1 (green), rabbit anti-occludin (red) followed by DAPI nuclear stain (blue). [B] Protein expression of E-cadherin and ß-catenin was detected using immunofluorescence microscopy (original magnification, ×63) with mouse anti-E-cadherin (green), rabbit anti-ß-catenin (red); and nuclei were counterstained with DAPI. [C] Actin filaments were detected using fluorescence microscopy (original magnification, ×63) with phalloidin (red) and nuclei were counterstained with DAPI (blue). Representative images captured from cross-section of the spheroids are shown. The bars indicate 10 µm.

**Figure 2:**
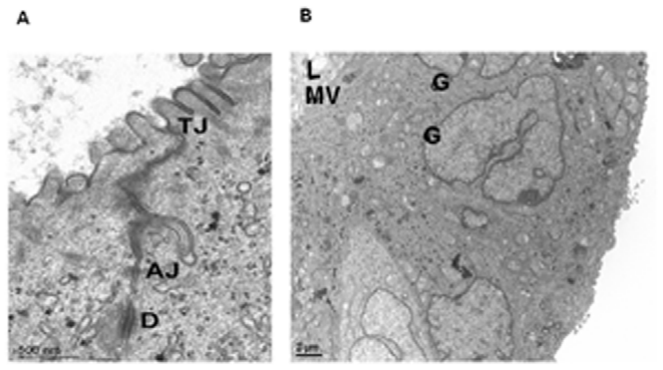
Transmission electron microscopy analysis of Caco-2 cells spheroids. A junctional complex (tight junction; TJ, adherens junction; AJ, D; desmosome) between adjacent cells is evident, apically [A]. The 3-D culture resulted in a luminal space (L) with formation of microvilli (mv) at the apical side and polarization of cells located in the cortical region of spheroid is indicated by supranuclear localization of Golgi (G) apparatuses [B].

After 7 days in culture, Caco-2 cells embedded in 40% Matrigel formed hollow multicellular spheroids consisting of a single layer of 40–50 cells and expressed the TJ proteins ZO-1 and occludin at the intercellular junctions surrounding the lumen (Figure 1A). The adherence junction (AJ) proteins, ß-catenin and E-cadherin were expressed basolaterally at the regions of cell-cell contact and at the basal side (Figure 1B). The luminal side of the cytoplasm of the cells in the spheroids was filled by actin filaments stained with the TRITC-conjugated phalloidin (Figure 1C). By TEM, well developed bundles of microvilli facing the lumen and the junctional complex (TJs, AJs, desmosomes) between cells could be distinguished (Figure 2A); the Golgi apparatus was localized at the supranuclear region of the Caco-2 cells (Figure 2B).

### Effects of Ethanol and Acetaldehyde on Paracellular Permeability

**Figure 3:**
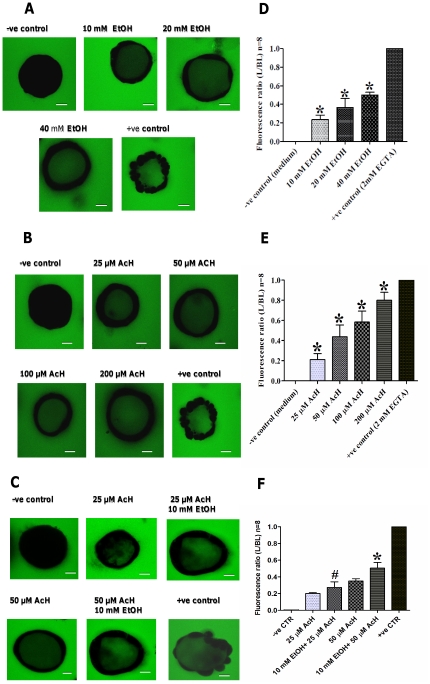
Ethanol and acetaldehyde and in combination increase permeation of the fluorescent marker FD4 from the basolateral to the luminal side of Caco-2 spheroids. [A–C] Spheroids were exposed at the basal side, in the presence of FD4, to medium only (−ve control), 2 mM EGTA (+ve control) and either 10 mM, 20 mM, 40 mM ethanol (panel A), 25 µM, 50 µM, 100 µM or 200 µM acetaldehyde (panel B) or in combination (panel C). Intraluminal accumulation of FD4 (green) was measured using confocal microscopy (original magnification, ×63) and representative images captured from the middle transsection of spheroids are shown and the bars indicate 10 µm. **Ethanol, acetaldehyde and in combination increase FD4 permeation dose-dependently in Caco-2 spheroids.** [D–F] The mean fluorescence intensity of FD4 from 8 spheroids was measured and expressed as the ratio of the luminal (L) over the basal (BL) compartment. The L/BL ratio of EGTA exposure (maximal TJ disruption) was set to 1. All graphs indicate the results of three replicate experiments. Data were expressed as means±SD, for ethanol (D), for acetaldehyde (E) and for combination (F), *P<.0001.

In unexposed spheroids, FD4 was exclusively observed in the culture medium/matrigel matrix at the basolateral side of spheroids (Figure 3A) resulting in a very low luminal to basolateral (L/BL) fluorescent ratio (Figure 3D). Exposure to EGTA (2 mM), which is well-known to disrupt tight junctions, resulted in a rapid FD4 flux (Figure 3A) and the L/BL ratio in this condition was set to 1 (Figure 3D–3F). Exposure of Caco-2 spheroids to ethanol (10–40 mM) increased the intraluminal FD4 fluorescent signals (Figures 3A) and significantly increased the L/BL fluorescent ratio, when compared to the unexposed spheroids, in a dose- dependent manner (Figure 3D). Similarly, exposure to acetaldehyde (25–200 µM) for 3 h increased the intraluminal FD4 fluorescent signals (Figures 3B) and significantly increased the L/BL fluorescent ratio when compared to the unexposed spheroids, in a dose-dependent manner (Figure 3E). Next, the effects of combined exposure to ethanol and acetaldehyde on barrier function were examined. As shown in Figure 3C, exposure to 25 µM or 50 µM acetaldehyde in the presence of 10 mM ethanol significantly increased the L/BL FD4 ratio versus exposure to (25 µM or 50 µM) acetaldehyde alone (Figure 3F).

### Effects of Ethanol and Acetaldehyde on Cell Viability

Exposure of Caco-2 spheroids to ethanol (10, 20 and 40 mM) and acetaldehyde (25, 50, 100 and 200 µM) did not significantly reduce cell viability (96.2±5.5, 99.0±5.4, 108.8±4.5; ethanol) and (90.4±1.7, 99.3±1.3, 99.1±1.5, 99.5±2; acetaldehyde), whereas exposure to 9% Triton X-100 solution significantly reduced cell viability towards 24.7±3.9% (compared to negative control; *P*<.0001), (data not shown).

### Effects of Ethanol and Acetaldehyde on Tight Junction Integrity and morphology

**Figure 4:**
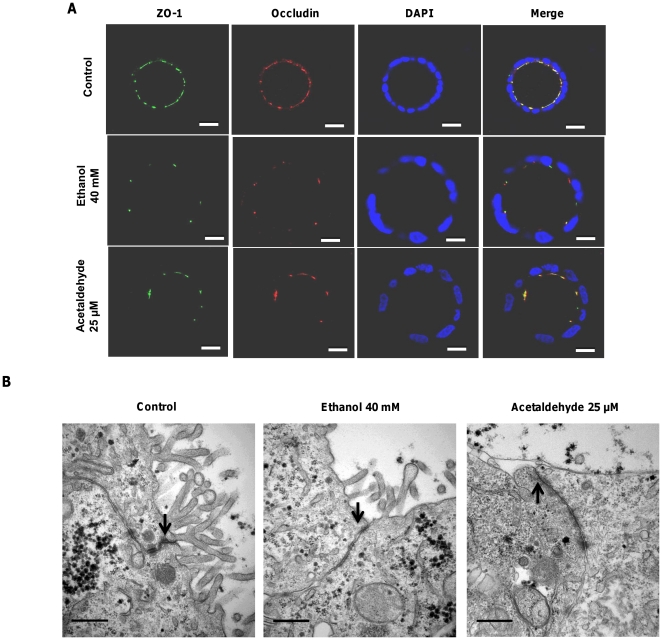
Ethanol and acetaldehyde exposure alters ZO-1 and occludin distribution at tight junctions of the Caco-2 spheroids. [A] Spheroids were exposed to medium only as control, 40 mM ethanol or 200 µM acetaldehyde for 3 h and labeled for ZO-1 (green), occludin (red) and nuclei (blue) by confocal immunofluorescence staining (original magnification, ×63) and representative images captured from the middle transsection of spheroids are shown. Bars indicate 10 µm. **Effects of ethanol and acetaldehyde on TJ morphology.** [B] Caco-2 spheroids were exposed to medium only, either ethanol or acetaldehyde for 3 h, fixed and processed for Transmission electron microscopy. The lateral surface is indicated by arrows. Bars indicate 0.5 µm.

As tight junctions are crucial for the paracellular barrier function, the effects of ethanol and acetaldehyde on the TJs integrity were determined. The 3 h exposure to ethanol (40 mM) and acetaldehyde (200 µM) resulted in loss of ZO-1 and occludin at the intercellular junctions as compared to the negative control (Figure 4A). Other concentrations of ethanol (10, 20 mM) and acetaldehyde (100 µM) had similar effects (data not shown). At the ultrastructural level, cell-cell junctions displayed no visible differences between control and ethanol- or acetaldehyde-treated spheroids (Figure 4B). A reduction or change in the appearance of microvilli was noted in acetaldehyde-treated cells (Figure 4B).

### Effects of Ethanol and Acetaldehyde on Protein Acetylation

**Figure 5:**
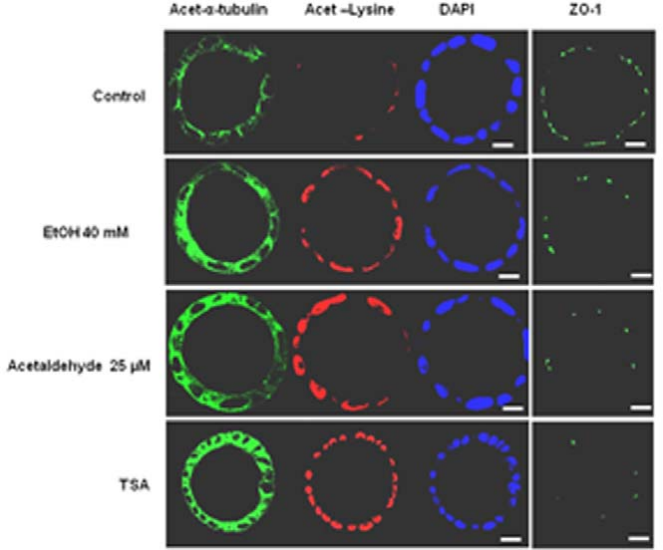
Hyperacetylation of α-tubulin and lysine residues, and localization of ZO-1 in Caco-2 spheroids. Protein expression of acetylated-α-tubulin, acetylated-lysine residues and ZO-1 detected using immunofluorescence microscopy (original magnification, ×63) with mouse anti-α-tubulin (green), rabbit anti-acetylated-lysine (bred), and nuclei were counterstained with DAPI (blue) and mouse anti-ZO-1 (green). Representative images captured from cross-sections of the spheroids are shown. The bars indicate 10 µm.

Since acetylation of microtubules and other proteins at lysine residues can affect different cell structures, possibly including the TJs, non-treated (negative control), ethanol, acetaldehyde and Trichostatin A (TSA; positive control)-treated spheroids were stained for acetylated α-tubulin, acetylated lysine residues, DAPI, and ZO-1. Ethanol, acetaldehyde, and TSA-treated spheroids showed an increased staining of acetylated α-tubulin and lysine residues compared to the control spheroids (Figure 5). Given the important role of the microtubules in TJ assembly, we investigated whether ethanol and acetaldehyde induced-α-tubulin hyperacetylation correlated with a disrupted ZO-1 localization pattern. Ethanol, acetaldehyde and TSA-treated spheroids showed a marked α-tubulin hyperacetylation associated with ZO-1 mislocalization when compared to the control spheroids (Figure 5).

### Effects of Ethanol and Acetaldehyde on Tight Junction Gene Expression

**Figure 6:**
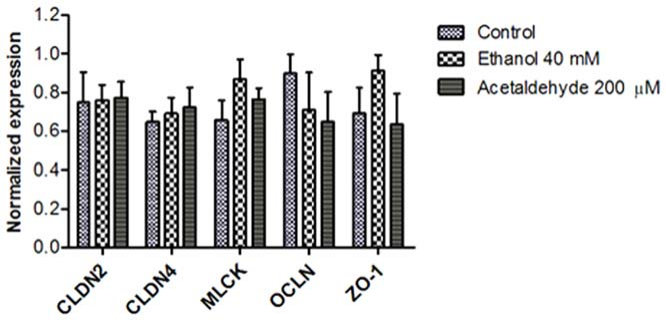
Ethanol and acetaldehyde did not alter expression of the tight junction-coding genes in Caco-2 spheroids. Spheroids were incubated with medium only (control), 40 mM ethanol and 200 µM acetaldehyde. Data were expressed as means of three replicates ± SD; all *P* values>0.05 comparing ethanol or acetaldehyde vs. control.

The mRNA expression of claudin 2, claudin 4, MLCK, ZO-1 and occludin did not reveal any differences in expression patterns between either ethanol or acetaldehyde-exposed and non-exposed Caco-2 spheroids (all *P*>.05 versus control, Figure 6).

## Discussion

In the present study, using a 3D Caco-2 cell culture model, ethanol as well as acetaldehyde at concentrations as seen after moderate ethanol ingestion dose-dependently increased paracellular permeability. The combined exposure resulted in an additive but not a synergistic effect. Furthermore, ethanol and acetaldehyde caused a redistribution and intracellular mislocalization of ZO-1 and occludin, and induced lysine residue and microtubule hyperacetylation. These changes were detected without a significant loss of cell viability or altered TJs-encoding gene expression.

Three-dimensional cell culture models are increasingly used to investigate epithelial pathophysiology. Since cell-stromal interaction is crucial in epithelial cell biology, a more realistic microenvironment is established to elicit an *in vivo* like response to stressors [44]. Therefore, the 3D cultures provide more physiological cellular interactions which are important for cell behavior, stability and gene expression. In contrast, epithelial cells grown as 2D monolayers on artificial membranes, polyethylene terephathalate membranes for example, may partially lose their original characteristics as the monolayer growth being largely influenced by different factors including the physicochemical properties of the membrane [45].

We and others have shown that Caco-2 cells are capable of differentiating into 3D spheroids with a central lumen that exhibit numerous features of intestinal epithelium *in vivo* [42], [46], [47]. This includes full polarization of the cells, development of tight and adherens junctions, and formation of microvilli [35], [41], [48].

In this study, the effects of ethanol and acetaldehyde at concentrations found in the blood after moderate ethanol consumption on barrier function using a 3D cell culture model were investigated. In moderate drinking the ethanol concentration in the blood can reach concentrations ranging from 5 to 6 mg/dl, to levels of over 90 mg/dl (∼20 mM) [49]. Furthermore, concentrations at 0.1% (∼20) and 0.2% (∼0 mM) ethanol have been used by others to gain mechanistic insight into ethanol-induced intestinal barrier dysfunction [50], [51], [52]. Therefore, the ethanol concentrations used in the present study are within the range of the blood concentrations, providing view of the changes caused by basal exposure of the intestinal epithelium to ethanol. Moderate ethanol consumption is defined as one standard drink (i.e. 12 grams of ethanol) a day for women, and two standard drinks a day for men [32], [53]. Exposure of the 3D Caco-2 spheroids at the basal side, mimicking *in vivo* basolateral exposure of the intestinal epithelium to concentrations of 10–40 mM ethanol, in the range of those found in the blood after moderate ethanol consumption, dose-dependently increased the paracellular permeability [43]. These observations indicate that ethanol at concentrations in the blood observed in moderate drinkers already may give rise to intestinal barrier dysfunction. Therefore, further studies investigating the mechanisms of moderate ethanol consumption-induced intestinal injury are warranted. Despite the morphological differences between 2D and 3D models, these results of the 3D model are in line with those obtained with the 2D model [21], [22]. However, most ethanol concentrations used in the 2D model were 20–80 fold higher (∼0.217–3.255 M) than those found in the distal small and large intestines after moderate ethanol consumption. The current data support recent observations that exposure to ethanol, at concentrations which can reach the distal small intestine as well as the colon, is able to disrupt the epithelial integrity *in vitro* [52], [54]. It is known that Caco-2 monolayers have tighter cell junctions and thereby are more resistant to noxious agents than the human small intestinal epithelium [55], [56]. This may explain why only luminal ethanol concentrations greater than 1% ∼217 mM were able to decrease the paracellular barrier function in Caco-2 monolayers. In this study, exposure of Caco-2 spheroids to (25–200 µM) acetaldehyde resulted in a dose-dependent increase in the paracellular permeability. Caco-2 spheroids were exposed at the basolateral side mimicking the exposure to acetaldehyde found in the blood. Serum concentrations of acetaldehyde depend on the amount of ethanol consumed and the presence of different alcohol dehydrogenases and aldehyde dehydrogenase isozymes [43]. In other studies, luminal exposure of Caco-2 monolayers to higher concentrations (100–600 µM) of acetaldehyde increased the paracellular permeability by disrupting the TJs integrity [23], [24], [25], [26], [27], [28]. Acetaldehyde was found more potent than ethanol in disrupting the barrier function, that is similar effects on permeability were reached at much lower concentrations. Based on these observations, combined exposure of the intestinal mucosa to these two barrier stressors *in vivo* will lead to more deleterious effects on the barrier function. Recently, Geetha *et al.*, have shown that exposure to ethanol at 100 mM did not increase the paracellular permeability in 2D Caco-2 monolayers, but in combination with 100 and 200 µM acetaldehyde synergistically increased the paracellular permeability through a Src kinase and MLCK-dependent mechanisms [29]. The differences between results may be related to the differences in the cell culture models and the permeability markers used.

Data from the present study revealed that neither ethanol nor acetaldehyde at the indicated concentrations compromised cell viability. Inspection of TJ and other cell-cell junctions at the ultrastructural level with electron microscopy revealed no apparent differences between control and ethanol- or acetaldehyde-exposed Caco-2 spheroids. However, in ethanol- or acetaldehyde-exposed Caco-2 spheroids, a reorganization of ZO-1 and occludin at the intercellular junctions and mislocalization of these proteins was found in conjunction with the observed enhancement in paracellular permeability. These data suggest that ethanol and acetaldehyde alter the molecular composition of TJ which likely affects TJ integrity. Our data contribute to the mounting evidence that loss of TJs integrity in response to both, ethanol and acetaldehyde is responsible for barrier dysfunction [21], [22], [23], [24]. It has been shown that luminal exposure of Caco-2 monolayers to ethanol (2.5 to 15% = 0.543–3.255 M) increased nitration of tubulin and disruption of barrier function by inducible nitric oxide synthase-dependent mechanism [22], [57]. Furthermore, ethanol (5–10% = 1.085–2.170 M) reduced TER of Caco-2 cell monolayers and increased paracellular permeability by a myosin light chain kinase-dependent mechanism [21]. Very recently, it has been shown that ethanol-induced intestinal hyperpermeability may require Snail activation via inducible nitric oxide synthase (iNOS) and p21-activated kinase (PAK1) [57]. Interestingly, Swanson and colleagues have shown that ethanol can induce intestinal hyperpermeability through stimulation of intestinal circadian clock gene expression [52]. Mechanisms of acetaldehyde-induced barrier dysfunction have largely been attributed to protein phosphatase and tyrosine kinase activation. Such increase in their activities can ultimately inhibit regulation of the phosphorylation-dephosphorylation balance of the TJ and AJ proteins and consequently, disruption of the barrier function [24], [27]. Ethanol has been shown to alter microtubule morphology in Caco-2 monolayers and consequently, barrier dysfunction [58]. Here, we have shown that Caco-2 spheroids basolaterally exposed to ethanol or acetaldehyde exhibit intense immunreactivity to antibodies against acetylated α-tubulin and lysine residues. Interestingly, simultaneous immunostaining revealed a concomitant α-tubulin hyperacetylation and ZO-1 mislocalization, suggesting that microtubule stabilization resulting from acetylation may lead to TJs disruption with subsequent increase in paracellular permeability. Trichostatin A mimicked the effect, suggesting that ethanol- and acetaldehyde-induced barrier dysfunction may be due to modulation of the TJs by histone acetylation. In line with this data, induction of histone acetylation has been shown to be associated with loss of TJs integrity [59]. In hepatocytes has revealed that α-tubulin is a major target for ethanol and acetaldehyde-induced acetylation and impaired microtubule polymerization [60], [61], [62]. Since microtubule acetylation is regulated by coordinated activities of acetyltransferases and the microtubule-specific deacetylases, sirtuin T2 (SirT2) and histone deacetylases 6 (HDAC6), it is suggested that ethanol and acetaldehyde treatment inhibits these deacetylases activity especially SirT2 due to its NAD^+^ dependency, leading to microtubule hyperacetylation [61], [63], [64], [65], [66]. The data show that hyperacetylation of microtubules is associated with ethanol-induced TJ disruption may further explain the molecular mechanism of ethanol-induced intestinal barrier dysfunction. In the present study, 3 hour exposure to either ethanol or acetaldehyde did not affect the TJs genes at the transcription level. However, Yueming Tang *et al.* demonstrated that 24 h exposure to ethanol (at 1% = 217 mM) upregulated microRNA (miR-212) expression, thereby down-regulating the translation of ZO-1 and increased the paracellular permeability of Caco-2 monolayers [50]. In a combined *in vivo* and *in vitro* study, ethanol exposure (5% vol/vol = 1.085 M) for 5 h decreased the mRNA levels of TJs proteins through dysfunction of hepatocytes nuclear factor-4α (HNF-4α) [67]. Since these studies have shown indirect effects of ethanol on TJ genes expression, further investigations are necessary to delineate the potential effects of ethanol and acetaldehyde on the expression of the TJ and AJ-encoding genes.

In summary, the results of the present study indicate that basolateral exposure of Caco-2 cells cultured in 3D to ethanol at concentrations found in the blood after moderate ethanol consumption, acetaldehyde and in combination, dose-dependently and additively increase paracellular permeability. Furthermore, ethanol and acetaldehyde induced lysine residue and microtubule hyperacetylation. This suggests that the changes on intestinal epithelial permeability may be induced, at least in part, by direct effects on the TJ and hyperacetylation of microtubules rather than loss of cell viability or altered TJ-encoding gene expression.

The 3D Caco-2 spheroids described in this study may represent a suitable cell culture model to study the intestinal epithelium under both, physiological and pathological conditions. Understanding the cellular mechanisms that regulate the intestinal epithelial barrier dysfunction induced by ethanol and its metabolites may lead to the development of therapeutic and/or nutritional strategies that are able to restore or prevent ethanol-induced intestinal epithelial damage during moderate ethanol consumption.
